# Postextractive Alveolar Ridge Preservation Using L-PRF: Clinical and Histological Evaluation

**DOI:** 10.1155/2020/5073519

**Published:** 2020-06-11

**Authors:** Giorgio Serafini, Marco Lollobrigida, Lorenzo Fortunato, Giulia Mazzucchi, Luca Lamazza, Dario Di Nardo, Iole Vozza, Mara Riminucci, Alberto De Biase

**Affiliations:** ^1^Department of Oral and Maxillo Facial Sciences, “Sapienza” University of Rome, Italy; ^2^Department of Molecular Medicine, “Sapienza” University of Rome, Italy

## Abstract

Leukocyte- and platelet-rich fibrin (L-PRF) is an autologous platelet concentrate rich in growth factors and plasma proteins, obtained by centrifugation of patient whole blood, and widely used in oral surgery. This report describes a case of alveolar ridge preservation with L-PRF membranes. Postextractive alveolar healing was then assessed through a histologic and histomorphometric analysis. A patient requiring tooth extraction and subsequent implant rehabilitation was treated with simple extraction and socket filling with L-PRF membranes. Implant placement was performed at 3 months, and a bone biopsy was obtained for histomorphometric analysis. Histological examination of the grafted sites showed that the use of L-PRF could achieve good results in terms of bone dimension and quality and soft tissue healing. The results of this study support the use of L-PRF membranes to preserve hard and soft tissues after tooth extraction.

## 1. Introduction

Several techniques have been described in the literature to maintain alveolar bone volume after tooth extraction and facilitate subsequent restorative procedures with endosseous implants.

Tooth extractions are common dental procedures used to remove decayed, damaged, or periodontally compromised teeth. Though associated with few adverse effects, most tooth extractions result in alveolar atrophy due to bone resorption and soft tissue remodeling. The most relevant alterations occur during the first three months after tooth loss, in particular in the buccal plate where bone is generally thinner. In a systematic review, Tan et al. [1] reported a 29-63% of horizontal and 11-22% of vertical bone loss during the first 6 months after tooth extraction without a ridge preservation technique. As a result, these volumetric changes often require bone augmentation techniques for correct implant placement.

Several techniques have been proposed to reduce bone resorption after tooth extraction. In particular, several bone substitutes (autografts, allografts, xenografts, or alloplastic materials) have been tested combined with resorbable or nonresorbable membranes [2, 3]. A recent systematic review [4] reported xenografts as the most beneficial for ridge preservation due to their resorption rate, followed by allografts and alloplastic materials when compared to natural healing. Though graft materials can reduce alveolar bone resorption, grafted sites often have poor vascularization and nonvital residual particles can negatively affect the bone to implant contact [5]. Furthermore, the exposure of resorbable or nonresorbable membranes, often used in combination with bone substitutes, could compromise bone regeneration and wound healing [6]. Finally, the high costs often limit the use of biomaterials in the clinical practice. In this context, autografts may represent an alternative but involve donor site morbidity and a rapid resorption [7].

New biologically active materials have been developed to overcome the disadvantages of conventional bone substitutes. These include first- and second-generation platelet concentrates, respectively, platelet-rich plasma (PRP) gel and leukocyte- and platelet-rich fibrin (L-PRF). L-PRF was described for the first time in France by Dohan et al. in 2006 [8] and includes both fibrin clots and liquid components [9]. L-PRF is obtained by centrifugation of patient whole blood without the addition of any additive or anticoagulants, like thrombin, calcium chloride, or EDTA. Once the clot is separated from the supernatant (Platelet Poor Plasma, PPP) and the red blood cells (Red Corpuscle Base, RCBs), it can be compressed into membranes. L-PRF membranes are composed of a dense, high cross-linked, fibrin mesh in which are embedded platelets and leucocytes. This biological scaffold releases growth factors (particularly, PDGF-AB, TGF-*β*, and VEGF), adhesion molecules (fibronectin, vitronectin, and thrombospondin-1), and pro- and anti-inflammatory cytokines, for up to 7 days [10, 11], which modulate reparative inflammatory process; increase tissue regeneration, angiogenesis, and neovascularization; and reduce postoperative pain and edema [12]. These characteristics make L-PRF suitable as grafting material for postextraction sockets, especially considering the modest costs, simple preparation, and no risk for cross infections. The aim of this study was then to report a case of postextraction socket grafted with L-PRF membranes through a clinical and radiographic evaluation after a 3-month healing period.

## 2. Case Report

A 35-year-old nonsmoker systemically healthy patient was referred to the Unit of Oral Surgery of the Department of Oral and Maxillo Facial Sciences, Sapienza University of Rome (Italy).

**Figure 1:**
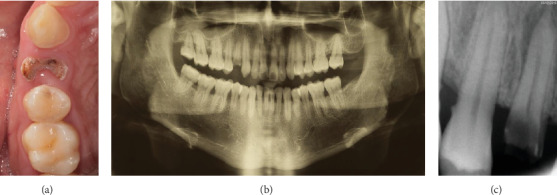
(a) Preoperative occlusal view; (b) orthopantomography; (c) preoperative periapical radiograph.

The intraoral exam revealed a nonrestorable carious lesion in the upper right first premolar (Figure 1(a)), confirmed with orthopantomography (Figure 1(b)) and periapical radiograph performed with Rinn holder (Figure 1(c)), in which a periapical periodontitis was highlighted. Before proceeding with tooth extraction, alternative therapeutic solutions, such as orthodontic extrusion or crown lengthening followed by endodontic therapy and prosthetic rehabilitation, were explained to the patient. Considering the advantages and disadvantages of these therapies, in agreement with the patient, the treatment plan included tooth extraction and socket grafting with L-PRF membranes from blood centrifugation, followed by a single unit implant rehabilitation.

The patient was treated in accordance with the Declaration of Helsinki of 1975, as revised in 2013, according to a specific protocol approved by the Ethics Committee for Human Research of Sapienza University of Rome (approval number: 981/17). A written informed consent was obtained from the patient before the treatment.

Seven days before tooth extraction, the patient was undergone scaling and root planing and trained to correct hygiene procedures.

**Figure 2:**
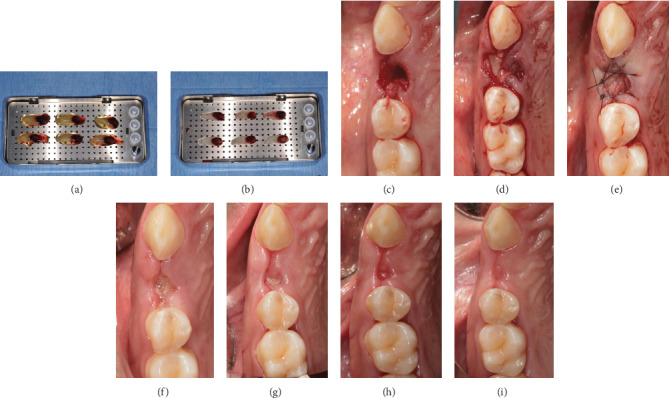
(a) L-PRF clots placed on a plate; (b) L-PRF membranes; (c) postextractive socket; (d) socket grafted with L-PRF membranes; (e) suture; (f) suture removal; (g) 14 days after tooth extraction; (h) 28 days after tooth extraction; (i) 60 days after tooth extraction.

On the day of the surgery, blood samples were collected from the patient in four tubes of 9 mL for a few minutes and immediately centrifuged. Centrifugation (Intra-Spin L-PRF kit, Intra-Lock® International Inc., Boca-Raton, Florida, USA) was performed for 12 minutes at 2700 rpm. Tubes did not contain any additive or anticoagulants. After centrifugation, L-PRF clots were collected from each tube and separated from the red thrombus (composed of red cells), obtaining a fibrin clot with a small red portion in order to include the intermediate portion rich in leucocytes and platelets [13]. L-PRF clots were then placed in a sterile box and slightly compressed for about 5 minutes under a stainless steel plate to form 4 membranes (Figures 2(a) and 2(b)).

Before surgery, the patient rinsed with 0.20% chlorhexidine for 1 minute and local anesthesia (2% mepivacaine with 1 : 100000 adrenaline) was administered. Atraumatic extraction was performed using a piezoelectric device (Piezosurgery®, Mectron, Genova, Italy) to preserve the buccal bone and avoid soft tissue laceration, followed by alveolar debridement and irrigation with sterile saline solution (Figure 2(c)). After tooth extraction, the buccal and palatal bone walls were both preserved. The patient had a thick biotype with a keratinized tissue width of 3 mm and buccal bone thickness of 1 mm. All the measures were recorded with a PCP-15-UNC probe (Hu-Friedy, Chicago, IL, USA). The socket was then filled with three L-PRF membranes previously folded and condensed with a sterile gauze (Figure 2(d)). Finally, one membrane folded in triple layer was placed to cover the socket. Soft tissues were sutured (4/0 nylon suture, 20 mm needle) at the mesial, median, and distal aspects of the socket, without primary closure (Figure 2(e)).

The patient was prescribed an analgesic/anti-inflammatory therapy (ketoprofen 100 mg or acetaminophen 1000 mg as needed), antiseptic mouth rinses (0.20% chlorhexidine twice daily for 7 days), and postoperative recommendations. The patient was advised to follow a soft and liquid diet, avoiding smoking and hot food for the following hours.

Sutures were removed at 7 days (Figure 2(f)), and the patient underwent a follow-up (14 days, 28 days, and 60 days) monitoring the wound healing (Figures 2(g)–2(i)).

Three periodontal indices (Plaque Index, PI; Gingival Index, GI; and Bleeding on Probing, BoP) were recorded on both the tooth to be extracted and the adjacent teeth the day of the surgery, at 14 days, 28 days, and 3 months in order to monitor the periodontal health conditions at the grafted site and adjacent teeth. According to Silness and Löe, PI was measured at 4 points (buccal, mesial, distal, and palatal) for each tooth [14]. According to Löe and Silness, GI was recorded to assess gingival inflammation through a visual evaluation with a value from 0 (healthy gum) to 3 (obvious inflammation and spontaneous bleeding) [15]. BoP (present or absent) was measured at 4 points (buccal, mesial, distal, and palatal) for each tooth [16]. After tooth extraction and before implant placement, the ridge width was measured at the median points of the alveolar crest in the buccal-palatal direction. A PCP-15-UNC probe was used for recording both alveolar and periodontal parameters. Alveolar ridge width in the buccal-palatal direction was 8 mm immediately after extraction and 7 mm at 3 months showing a minimal reduction. The numerical reduction in periodontal indices during the follow-up indicates a reduction of gingival inflammation (Table 1), which can be explained with an increased compliance of the patient with postoperative hygiene instructions and, as for the 14-dayfollow-up, possibly also due to the antimicrobial action of leukocytes.

**Figure 3:**
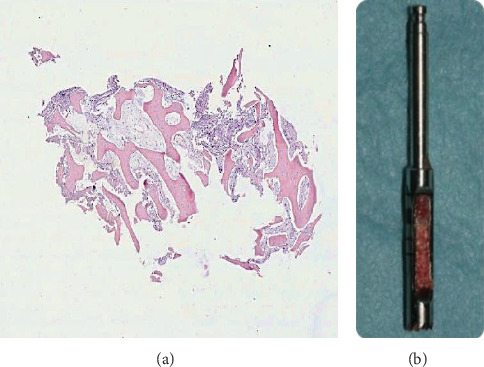
Histological specimen of the bone tissue taken at the center of the grafted site showing bone tissue surrounded by connective tissue matrix. Hematoxylin-eosin staining; (a) original magnification ×5; (b) surgical trephine bur with bone tissue sample.

After 3 months, before implant placement, a bone biopsy was performed with a surgical trephine bur (2.0 mm internal diameter) at the center of the ridge for histomorphometric analysis of the grafted site (Figures 3(a) and 3(b)) according to Corsi et al. [17]. The tissue specimen was fixed in 4% neutral buffered formaldehyde for 24 hours at 4°C. After fixation, the sample was repeatedly washed in phosphate buffer (pH 7.0), decalcified with 4% EDTA in phosphate buffer (pH 7.0) at 4°C for 48 hours, and routinely embedded in paraffin. Sections of 3 *μ*m thickness were cut from paraffin blocks and used for hematoxylin-eosin stain. The analysis of the bone sample showed newly formed trabecular bone in the grafted site. The trabecular architecture of the bone sample indicates a physiological healing of the postextraction site. At histomorphometric analysis, the bone tissue with osteocytes was 41.8% while the remaining part was composed by connective tissue.

**Figure 4:**
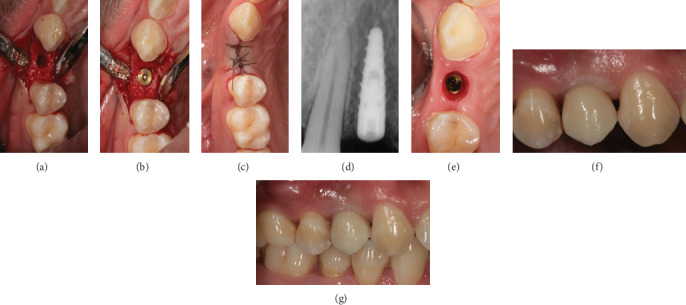
(a) Implant site preparation; (b) implant placement; (c) suture; (d) periapical radiograph after implant placement; (e) soft tissue conditioning; (f) screw-retained zirconia crown; (g) follow-up 6 months after final restoration.

After bone biopsy (Figure 4(a)), a 3.7 × 10 mm implant (Intra-Lock®, Boca Raton, Florida, USA) was placed (Figure 4(b)) and soft tissues sutured (4/0 nylon suture, 20 mm needle) with primary closure (Figure 4(c)). A periapical radiograph was also performed at this time to check correct implant placement (Figure 4(d)). Sutures were then removed 7 days after implant surgery, and a healing abutment was positioned during the second surgery two months later to obtain soft tissue conditioning (Figure 4(e)). A silicone impression with individual tray was taken one month later to fabricate a screw-retained zirconia crown (Figure 4(f)). After 6 months from final restoration, the patient came back for a follow-up, which revealed a stability of the peri-implant soft tissues.

## 3. Discussion

In the present study, a patient requiring tooth extraction and implant rehabilitation was treated with simple extraction and socket filling with L-PRF membranes. Implant placement was performed three months after ridge preservation, and a bone biopsy was obtained for histomorphometric analysis.

Maintaining an adequate alveolar ridge volume is essential for successful implant placement. However, several internal and external changes of the extraction socket occur after tooth extraction, in particular in the buccal plate, causing a loss of hard and soft tissues [18].

Alveolar ridge preservation includes several techniques to reduce the dimensional changes of hard and soft tissues following tooth extraction. During the last years, several techniques have been proposed [18]. Though graft materials were proven to reduce alveolar bone resorption, they often have poor vascularization and less vital bone formation compared to spontaneous healing [6]. Furthermore, the exposure of resorbable or nonresorbable membranes, often used in combination with bone substitutes, could compromise bone regeneration and wound healing [6]. Finally, the high costs often limit the use of biomaterials in the clinical practice.

Leukocyte- and platelet-rich fibrin (L-PRF) was first described by Dohan et al. in 2006 [8]. It is considered a second-generation platelet concentrate obtained by centrifugation of patient whole blood without the addition of any additive or anticoagulants. With this kind of preparation technique, at least 95% of platelets are embedded into the fibrin network of L-PRF [19]. High concentrations of platelets allow the slow release of growth factors from their alpha granules [20]. These growth factors include Platelet-Derived Growth Factor (PDGF), Vascular Endothelium Growth Factor (VEGF), Transforming Growth Factor-beta (TGF-beta), Fibroblast Growth Factor (FGF), Epidermal Growth Factor (EGF), Insulin-like Growth Factor (IGF), and Hepatocyte Growth Factor (HGF). All of these play an important role in wound healing, in particular improving hemostasis, angiogenesis, and epithelialization. L-PRF has been then used in several surgical procedures, such as oral and maxillofacial surgery and periodontal surgery [21], in order to enhance wound healing process.

The results of this report are consistent with those found in other studies [22, 23], where L-PRF showed to be effective in preserving hard and soft tissues without interfering with physiological bone healing process. Ridge preservation techniques are always desirable when functional and aesthetic results are demanded. Such procedures also facilitate implant placement according to the prosthetic project and with a proper macrogeometry. The beneficial effects of L-PRF in reducing dimensional changes may derive from its capacity to promote tissue regeneration. The dimensional remodeling observed in this study is comparable to those reported with other techniques [24] where bone substitutes were used. However, a recent randomized controlled trial [25] has suggested that PRF may not significantly enhance bone formation after tooth extraction, as compared to spontaneous healing. Even though studies with larger population will be required to reach valid conclusions, current results may provide the base to investigate the combined use of PRF and bone substitutes. Notwithstanding, differently from other techniques, the use of L-PRF is relatively simple and requires minimal costs, no need for primary closure and no risk for membrane exposure. In addition, as an autologous product, there is no risk of disease transmission or graft infection.

Within the limitations of this report, our results support the use of L-PRF in alveolar ridge preservation when implant placement is scheduled after tooth extraction. Additional studies with a larger sample size, randomization, and a split-mouth design are needed to confirm our findings and investigate possible specific indications of L-PRF compared to other biomaterials.

## Figures and Tables

**Table 1:** Periodontal index measures.

Periodontal indices		Baseline			14 days		28 days		3 months	
		1.3	1.4	1.5	1.3	1.5	1.3	1.5	1.3	1.5
GI		1	1	1	1	0	0	0	0	0

PI	B	2	2	2	1	1	0	0	0	0
	M	1	1	1	1	1	0	0	0	0
	D	1	1	1	1	1	1	0	1	0
	P	2	2	2	1	1	1	1	0	0

BoP	B	-	+	-	-	-	-	-	-	-
	M	-	+	+	-	+	-	-	-	-
	D	+	+	+	+	+	-	+	-	-
	P	-	+	+	-	-	-	-	-	-

GI: Gingival Index; PI: Plaque Index; BoP: Bleeding on Probing; B: buccal; M: mesial; D: distal: P: palatal.
